# Systematic Review of Patient and Caregiver Involvement in CKD Research

**DOI:** 10.1016/j.ekir.2025.03.018

**Published:** 2025-03-17

**Authors:** Talia Gutman, Dale Coghlan, Jonathan C. Craig, Chandana Guha, Allison Jaure, Shilpanjali Jesudason, Adeera Levin, David M. White, Javier Recabarren Silva, Anita van Zwieten, David Tunnicliffe, Andrea K. Viecelli, Germaine Wong, Armando Teixeira-Pinto, Siah Kim, Stephen McDonald, Carmel M. Hawley, Nicole Scholes-Robertson

**Affiliations:** 1 Sydney School of Public Health, Faculty of Medicine and Health, The University of Sydney, Sydney, New South Wales, Australia; 2 College of Medicine and Public Health, Flinders University, Adelaide, South Australia, Australia; 3 Centre for Kidney Research, The Children’s Hospital at Westmead, Sydney, New South Wales, Australia; 4 Central Northern Adelaide Renal and Transplantation Service, Royal Adelaide Hospital, Adelaide, South Australia, Australia; 5 School of Medicine, Faculty of Health and Medical Sciences, University of Adelaide, South Australia, Australia; 6 The University of British Columbia, Vancouver, British Columbia, Canada; 7 American Association of Kidney Patients, US Food and Drug Administration Patient Engagement Advisory Committee; 8 Department of Kidney and Transplant Services, Princess Alexandra Hospital, Brisbane, Queensland, Australia; 9 Australia and New Zealand Dialysis and Transplant Registry, South Australia Health and Medical Research Institute, Adelaide, South Australia, Australia; 10 Australasian Kidney Trials Network, Faculty of Medicine, University of Queensland, Brisbane, Queensland, Australia; 11 Faculty of Medicine, University of Queensland, St Lucia, Queensland, Australia; 12 Metro South Kidney and Transplant Services, Princess Alexandra Hospital, Woolloongabba, Queensland, Australia

**Keywords:** chronic kidney disease, consumer involvement, patient involvement, research collaboration

## Abstract

**Introduction:**

Limited consumer involvement in chronic kidney disease (CKD) research may reduce its relevance, impact, and transferability into practice and policy. We aimed to describe the current landscape of consumer (patients with CKD and caregivers) involvement in published CKD research.

**Methods:**

Electronic databases were searched to August 2023. Articles describing consumer involvement in CKD research were eligible. All text were imported into NVivo for line-by-line coding using descriptive synthesis of these domains: defining involvement, purpose of involvement, selection, stages of the research, resources, and evaluation.

**Results:**

We included 106 articles that involved over 4500 consumers from 15 countries. Eighty-two articles (77%) defined consumer involvement, using 8 different terms. Forty-three articles (41%) addressed reasons for involving consumers in research. Consumers were predominantly identified through clinical or patient networks based on demographic or clinical characteristics. Those involved at higher levels (e.g., coresearcher/patient partner) often had medical or academic training. Consumers were rarely drivers or commissioners of research (*n* = 6, 6%) and were most likely to be involved as informants (*n* = 81, 76%) with limited decision-making power. Most articles described consumer involvement in priority setting (*n* = 48, 45%) and research design (*n* = 57, 53%) with less evidence of involvement in implementation (*n* = 28, 26%) and evaluation (*n* = 24, 22%). Barriers included limited resources (i.e., financial, logistical, or training) and the need for tailored solutions continue to exist. Consumer involvement resulted in increased recruitment and retention, richer data, and more useful outputs for end users.

**Conclusions:**

Consumers were mostly involved in discrete activities with limited decision-making power. Increasing financial, logistical, and training resources for consumers may support more meaningful involvement. Ongoing evaluation of processes or impacts of consumer involvement, including consistent reporting, is needed to strengthen evidence and practice in CKD research.

## Graphical abstract

**Figure:**
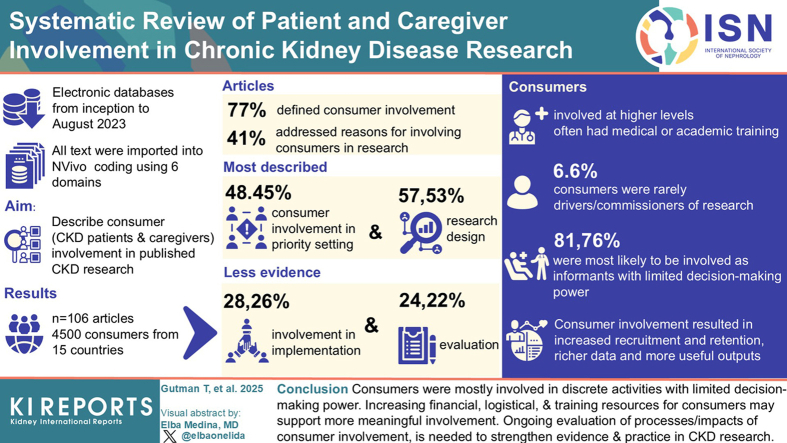

See Commentary on Page 1619

The misalignment of priorities between researchers and consumers (defined as patients and informal caregivers or family members) and the lack of involvement of consumers in research may limit the relevance and impact of research.^1^ It is estimated that 80% of clinical research in CKD does not address the top 10 research priorities identified by patients.^2^ In addition, outcomes consistently prioritized by consumers, such as mortality and fatigue, are infrequently reported in trials.^1^, ^2^, ^3^, ^4^, ^5^, ^6^, ^7^, ^8^

Consumer involvement in research has been referred to as participatory research, which is an approach that emphasizes the active engagement of patients, carers, and community members in the research process. This approach shifts from research conducted “on” people to research conducted “with” and “for” them, promoting cocreation, shared decision-making, and mutual benefit.^9^ Consumer involvement can help to improve recruitment, increase the acceptability and uptake of study findings, and impact on care and patient outcomes.^4^, ^5^, ^6^, ^7^ In recent years, there has been greater recognition and effort to involve consumers in research in nephrology; however, there remains uncertainty as to the approaches used.^10^

Reports of consumer involvement in research in CKD are generally lacking.^7^^,^^11^, ^12^, ^13^, ^14^ There remains uncertainty about best practices of involving patients or caregivers in research.^4^^,^^6^^,^^12^^,^^15^^,^^16^ Thus, a better understanding of successful processes and strategies for involving consumers in CKD research is needed. The review aims to ascertain how consumers were defined in the articles, how they were identified, their purpose of involvement, what stages of research they were engaged in, and how they were involved in these stages. A more comprehensive understanding of current practice may inform ways to strengthen consumer involvement in future research.

## Methods

**Figure 1:**
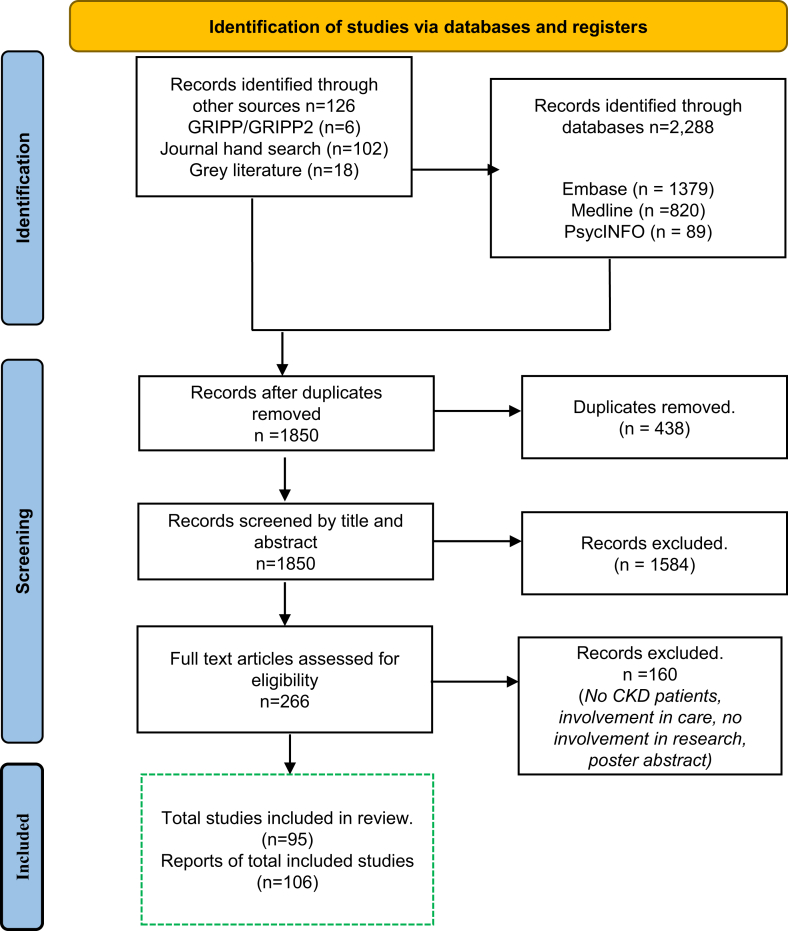
Systematic search results.

We used the preferred reporting items for systematic reviews and meta-analyses checklist (where possible) and flow diagram (Figure 1) to report this study.^3^^,^^17^, ^18^, ^19^, ^20^

### Literature Search

#### Selection Criteria

Articles that described the involvement of patients with CKD (all stages) and caregivers in any stage of the research process and for any type of health research or other publication (i.e., editorials, commentaries, and protocols) were eligible. Articles that were not published in English were excluded because of insufficient resources for translation. Consumer involvement was defined as any research activity that involved 1 or more consumers in any aspect or stage of the project.^21^ Publications were excluded if they involved patients or caregivers as research participants only. We used predefined inclusion and exclusion criteria based on established frameworks, including the INVOLVE framework and the National Framework for Consumer Involvement in Cancer Control.^3^^,^^22^ These criteria are presented in Table 1.^23^, ^24^, ^25^, ^26^, ^27^, ^28^, ^29^, ^30^, ^31^, ^32^, ^33^, ^34^, ^35^, ^36^, ^37^, ^38^, ^39^, ^40^, ^41^, ^42^, ^43^, ^44^, ^45^, ^46^, ^47^, ^48^, ^49^, ^50^, ^51^, ^52^, ^53^, ^54^, ^55^, ^56^, ^57^, ^58^, ^59^, ^60^, ^61^, ^62^, ^63^, ^64^ Articles were included if they described consumer involvement in any of these roles to allow for the assessment of the extent and nature of consumer contributions. We excluded articles that involved consumers as coauthors but did not describe any process for involving consumers in the main text. Although coauthorship may imply meaningful involvement, the lack of details regarding their involvement in these studies precluded their inclusion in our review. Without clear descriptions of the patient or caregiver involvement, it is challenging to determine the extent and nature of consumer contributions. This approach provided a structured and transparent method for evaluating the extent and nature of consumer contribution*s.* Reviewers DC, TG, and NSR independently screened the search results.

**Table 1:** Consumer roles and responsibilities

Role^a^	Definition^a^	Example responsibilities
Attendees	Receive information from researchers/experts with limited opportunity to contribute (i.e., educational seminar, information session)^23^, ^24^, ^25^	Participate in online forums. Attend meetings, conferences
Informants	Provide personal perspectives/experiences of condition/treatment/care (i.e., surveys, interviews, focus groups),^25^, ^26^, ^27^, ^28^, ^29^, ^30^, ^31^, ^32^, ^33^, ^34^, ^35^, ^36^, ^37^, ^38^, ^39^, ^40^, ^41^, ^42^, ^43^, ^44^, ^45^, ^46^, ^47^	Relay and relate personal experiences, provide testimony (regulatory) (e.g., managing fluid intake, usefulness of devices) Identify and prioritize (rate/rank) research questions, outcomes, topics. Provide preferences (e.g., aims, topics, formats, outcomes, trade-offs)
Advocates	Represent broad range of perspectives/experiences (i.e., patient organizations, patient representatives)^30^^,^^32^^,^^43^^,^^48^, ^49^, ^50^, ^51^, ^52^, ^53^, ^54^	Provide guidance around cultural safety and sensitivity, diversity Apply personal experiences to broader community/population needs Liaise between research and consumer communities
Advisors	Provide advice based on experiential knowledge (e.g., advisory group)^23^^,^^24^^,^^26^^,^^34^^,^^39^^,^^43^^,^^46^, ^47^, ^48^, ^49^^,^^51^^,^^52^^,^^55^, ^56^, ^57^, ^58^, ^59^, ^60^	Attend meetings/phone calls/teleconferences Monitor and review progress Provide feedback—written/verbal (e.g., search terms, protocols, recruitment/enrolment strategies) Advise on communication strategies with participants/consumers Provide advice and guidance on recruitment strategies Provide feedback on study/patient materials (e.g., interview guides, educational materials/sessions, support tools, consent forms, lay summaries, newsletters, progress reports, cultural messages)
Experts	Provide high quality knowledge and expertise as end user (i.e., board member with voting rights)^11^^,^^26^^,^^29^^,^^32^^,^^37^^,^^38^^,^^41^^,^^49^^,^^50^^,^^56^^,^^61^	Attend meetings/phone calls/teleconferences Alert research team to potential risks, ethical issues. Provide advice, insights, and guidance on implementation (e.g., core outcomes) Participate in strategic and practical decision making.
Partners	Participate as full members of the research team (i.e., coresearcher)^11^^,^^24^^,^^25^^,^^27^^,^^28^^,^^34^^,^^36^^,^^43^, ^44^, ^45^^,^^56^, ^57^, ^58^, ^59^, ^60^^,^^62^^,^^63^	Attend meetings/phone calls/teleconferences Connect the research team with the greater consumer community (e.g., community coordinator, consumer liaison, buddy) Contribute to study design/methods (e.g., study question, outcome selection, outcome measures, recruitment materials, power calculations, data collection tools, intervention design) Conduct interviews Contribute to/conduct analysis, interpretation and presentation of results (e.g., qualitative analysis, validation of themes, contextualizing quantitative data) Write recommendations Disseminate research findings (e.g., present at meetings/conferences, write papers for patient/academic publications)
Drivers	Lead, propose and drive research projects/agendas (i.e., patient led research network)^64^	Apply for grants, funding Identify project aims and objectives, define scope Monitor and review progress Govern networks/bodies/organizations (i.e., identify and prioritize research questions, allocate funding) Conceptualize, design, and implement sub studies (e.g., PROM sub study)

a Adapted from National Framework for Consumer Involvement in Cancer Control, Cancer Australia 2011.^3^

#### Search Strategy

We searched MEDLINE, Embase and PsycINFO, from inception to August 2023 using Medical Subject Headings and keywords for CKD and consumer involvement^65^ (Supplementary Table S1). We first searched for terms relating to CKD and then combined these with an existing validated search filter for terms related to consumer involvement^65^ (Supplementary Table S1). We supplemented our search by examining all selected articles that cited the Guidance for Reporting Involvement of Patients and Public (GRIPP)^19^ or GRIPP2^20^ checklists because they offer a structured approach to reporting patient and public involvement in research. We also searched journals on consumer involvement (e.g., Research Involvement and Engagement, The Patient), and websites of peak consumer organization for articles related to consumer involvement in CKD research (Supplementary Table S2). Screening was conducted in duplicate by 2 reviewers independently. Discrepancies were resolved through discussion, and when necessary, consultation with a third reviewer. This process ensured that the rigorous selection of articles aligned with the review’s objectives.

### Data Extraction and Synthesis Framework

We imported the completed text of these articles into NVivo software (release 1.7; QSR International, 2022) for analysis using a standard form (Supplementary Table S3). The initial domains were established *a priori* based on existing frameworks, including the Patient-Centered Outcomes Research Institute framework, the GRIPP and GRIPP2 checklists, the INVOLVE framework, and the National Framework for Consumer Involvement in Cancer Control.^3^^,^^18^, ^19^, ^20^, ^21^ Using descriptive synthesis, authors DC and TG synthesized existing frameworks and toolkits and selected the domains and categories that are most important for assessing consumer involvement.^3^^,^^18^, ^19^, ^20^, ^21^ Regular meetings were held to review and refine the coding framework, ensuring consistency and accuracy across all articles. We coded the selected articles according to the following domains: defining involvement (definitions for involvement, involvement activities, and levels of involvement); stating the purpose of involvement (alignment with project aims and purposeful involvement); selecting consumers (selection number, type, representation, and roles); stages of the research (priority setting, design, data collection, data analysis, dissemination, implementation, and evaluation); resources (financial and training or education); and evaluation of impact (challenges, benefits, and impacts).^3^^,^^18^, ^19^, ^20^, ^21^

## Results

### Search Results and Study Characteristics

We included 106 articles that involved 4400 consumers (including 2697 patients and 580 caregivers), from the following 15 countries: Australia (*n* = 36 publications), Canada (*n* = 29), USA (*n* = 20), UK (*n* = 14), Netherlands (*n* = 5), Denmark (*n* = 4), France (*n* = 2), Ireland (*n* = 1), China (*n* = 1) Korea (*n* = 1), Germany (*n* = 1), Italy (*n* = 1), Spain (*n* = 1), Guatemala (*n* = 1), and Sweden (*n* = 1) (Figure 1, Table 2). With the current search strategy, there were no randomized controlled trials identified, although several papers (*n* = 19.18%) included in this review informed the design, core outcomes, and interventions for clinical trials or observational studies.^26^, ^27^, ^28^^,^^48^^,^^55^^,^^66^, ^67^, ^68^, ^69^, ^70^, ^71^, ^72^, ^73^, ^74^, ^75^ All articles were published since 2008; however 72 (68%) were published since 2019. Four articles (4%) used the GRIPP and GRIPP2.^29^^,^^62^^,^^76^^,^^77^

**Table 2:** Characteristics of included articles (*n* = 106)

Characteristic	*n* (%)
Year published	o
< 2008	1 (1%)
2009–2013	7 (7%)
2014–2018	26 (25%)
2019–2023	72 (68%)
Number of consumers included^a^	
1–10	43 (41%)
11–30	24 (23%)
31–100	12 (11%)
101–200	5 (5%)
> 200	6 (6%)
Not reported/applicable	16 (15%)
Country	
Australia	36 (34%)
Canada	29 (27%)
United States	20 (19%)
United Kingdom	14 (13%)
Other^b^	19 (18%)
Consumer roles	
Attendees	16 (15%)
Informants	81 (76%)
Advocates	36 (34%)
Advisors	64 (60%)
Experts	36 (34%)
Partners	74 (70%)
Drivers	6 (6%)
Stage of the research cycle	
Priority setting	48 (45%)
Design	57 (53%)
Data collection	29 (27%)
Data analysis	26 (24%)
Dissemination	31 (29%)
Implementation	28 (26%)
Evaluation	24 (22%)

a Consumer numbers not comprehensively reported, numbers indicate minimum number of consumers involved.

b Denmark, France, Germany, Italy, Spain, Ireland, Korea, Netherlands, Sweden, Guatemala, China, International.

### Descriptive Results

In the following paragraphs we use the coding framework to describe the involvement of consumers in published CKD research. Where possible, for each of the domains, we report the number and proportion of included articles (*N* = 106) that addressed the domain, describe the relevant reported consumer involvement actions, and summarize author reflections and recommendations.

#### Terms and Definitions of Involvement

**Figure 2:**
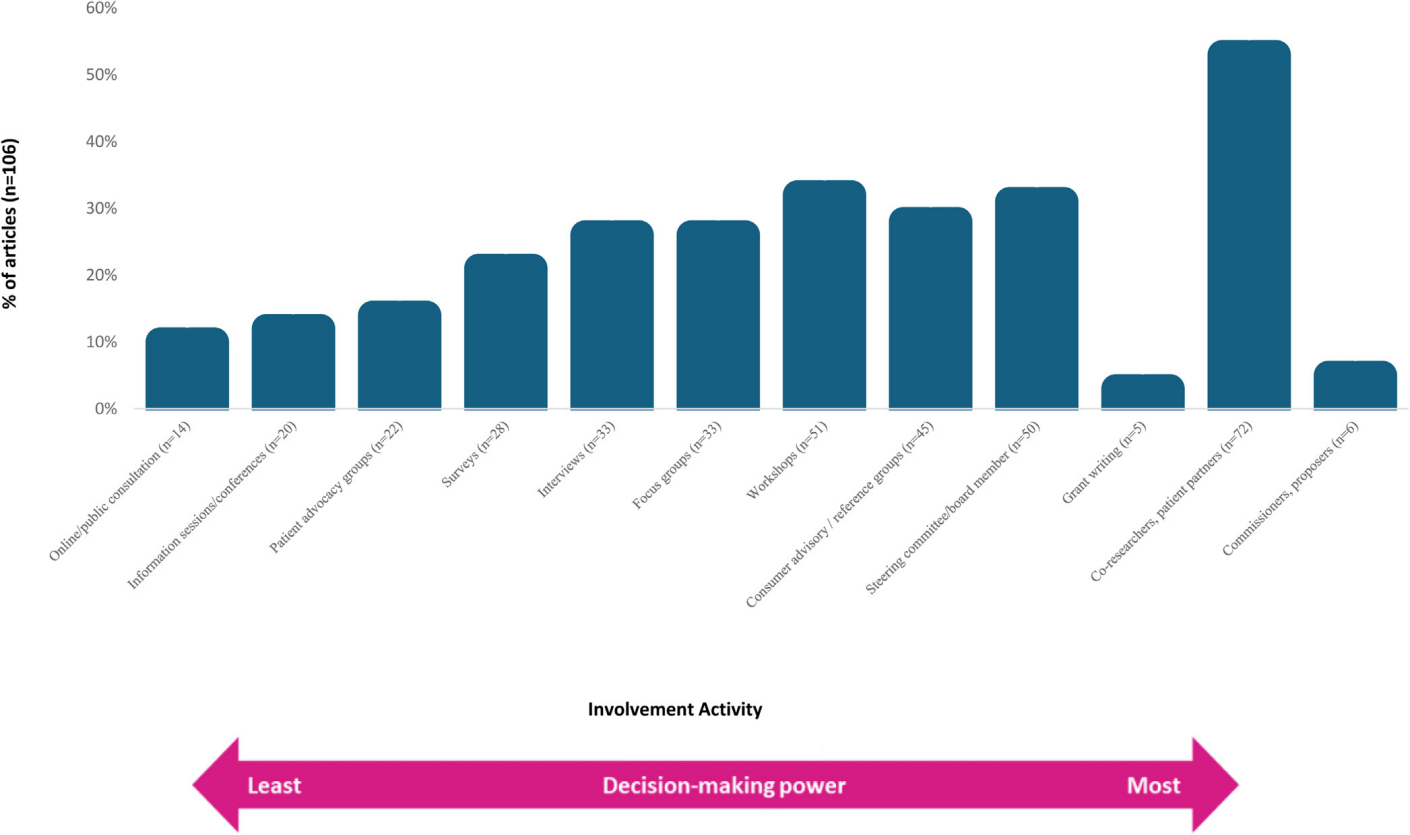
Involvement activities.

Across 82 articles (77%), the following 8 terms of consumer involvement were used: community-based participatory research, patient-researcher partnership, patient engagement, patient/consumer/public involvement, coresearcher, coproduction, patient partner (Supplementary Table S4). Fifteen articles provided definitions for 8 consumer involvement terms (Supplementary Table S4). Six articles referenced the INVOLVE “Research Cycle” definition.^30^, ^31^, ^32^^,^^56^^,^^78^, ^79^, ^80^, ^81^, ^82^ Some projects involved consumers in a single activity (e.g., priority setting workshops),^30^, ^31^, ^32^^,^^82^ whereas others involved consumers in multiple activities across the research process (e.g., consumer partners involved in all activities).^11^^,^^23^^,^^24^^,^^33^^,^^34^^,^^55^^,^^56^^,^^63^ The spectrum of reported consumer involvement activities and relationship to level of decision-making power are shown in Figure 2.

#### Stating the Purpose of Involvement

In total, 43 articles (41%) addressed the reasons for involving consumers in the research and provided general reasons, including improved relevance, importance, quality, knowledge translation, public accountability, and improved health outcomes.^10^^,^^26^^,^^29^, ^30^, ^31^^,^^33^, ^34^, ^35^, ^36^, ^37^, ^38^, ^39^, ^40^^,^^57^^,^^62^^,^^67^^,^^68^^,^^73^^,^^74^^,^^76^, ^77^, ^78^, ^79^, ^80^, ^81^, ^82^, ^83^, ^84^, ^85^, ^86^, ^87^, ^88^, ^89^, ^90^, ^91^, ^92^, ^93^, ^94^, ^95^, ^96^, ^97^, ^98^, ^99^ Twenty-eight articles (26%) involved consumers to identify and/or prioritize topics and outcomes for research.^30^^,^^31^^,^^34^^,^^37^^,^^40^^,^^66^^,^^68^^,^^71^^,^^72^^,^^77^^,^^78^^,^^80^^,^^82^^,^^85^^,^^87^^,^^88^^,^^90^^,^^92^^,^^95^^,^^99^, ^100^, ^101^, ^102^, ^103^, ^104^, ^105^, ^106^, ^107^, ^108^ Eight papers (8%) described project-specific purposes, including eliciting patient-specific knowledge, solutions, feedback, and advice; developing consumer materials, involvement strategies, and products; and generating consensus on critically important outcomes.^29^^,^^33^^,^^35^^,^^36^^,^^38^^,^^57^^,^^62^^,^^109^

#### Identifying and Selecting Consumers

Sixty-two publications (58%) described specific methods and settings for identifying consumers which included hospitals or clinics^23^^,^^28^^,^^33^^,^^41^^,^^42^^,^^49^^,^^62^^,^^69^^,^^73^^,^^74^^,^^82^^,^^89^^,^^93^^,^^110^^,^^111^; patient organizations or advocacy groups, committees, or charities^11^^,^^24^^,^^28^^,^^34^^,^^36^^,^^37^^,^^42^^,^^43^^,^^49^^,^^82^^,^^83^^,^^91^; social media^24^^,^^70^^,^^86^^,^^98^; established research networks and professional societies^24^^,^^28^^,^^36^^,^^37^^,^^67^^,^^71^^,^^78^^,^^79^^,^^85^, ^86^, ^87^, ^88^^,^^90^^,^^94^, ^95^, ^96^^,^^98^^,^^102^^,^^108^^,^^112^^,^^113^; posters, flyers, and emails^10^^,^^23^^,^^30^^,^^62^^,^^70^^,^^77^^,^^80^^,^^99^; health care professionals or researchers^11^^,^^30^^,^^33^^,^^49^^,^^68^^,^^70^^,^^82^^,^^114^^,^^115^; study participants (e.g., for embedded substudy)^29^^,^^44^^,^^63^^,^^104^; clinical or consumer registries and databases^75^^,^^76^^,^^103^^,^^105^, ^106^, ^107^^,^^116^^,^^117^; and known consumer research partners or leaders.^11^^,^^58^^,^^92^

#### Criteria and Considerations for Selection

Some articles involved consumers with experience in research (*n* = 13, 12%),^10^^,^^11^^,^^32^^,^^49^^,^^63^^,^^79^^,^^96^^,^^110^^,^^113^^,^^117^, ^118^, ^119^, ^120^ with a health or academic background (*n* = 9, 8%),^34^^,^^56^^,^^63^^,^^88^^,^^95^^,^^112^^,^^117^^,^^119^^,^^120^ who held advocacy roles (*n* = 16, 15 %),^10^^,^^32^^,^^50^^,^^74^^,^^78^^,^^79^^,^^83^^,^^85^^,^^87^^,^^89^^,^^90^^,^^104^^,^^113^^,^^118^, ^119^, ^120^ or consumers with whom researchers had an existing relationship (*n* = 11, 10%).^10^^,^^11^^,^^49^^,^^78^^,^^79^^,^^85^^,^^90^^,^^95^^,^^96^^,^^113^^,^^119^ Others sought consumers with no connection to the research team (*n* = 6, 6%),^49^^,^^62^^,^^69^^,^^83^^,^^92^^,^^117^ and/or little to no previous experience in research (*n* = 2, 2%).^44^^,^^45^ Some targeted consumers with specific experience of a treatment or disease (*n* = 19, 18%),^23^^,^^31^^,^^33^^,^^38^^,^^41^^,^^42^^,^^62^^,^^67^^,^^75^, ^76^, ^77^, ^78^^,^^89^^,^^90^^,^^92^^,^^97^^,^^104^^,^^112^^,^^118^ who were from disadvantaged and vulnerable groups (*n* = 10, 9%),^35^^,^^43^^,^^49^^,^^58^^,^^78^^,^^87^^,^^90^^,^^92^^,^^99^^,^^113^ those who spoke languages other than the country’s official language (*n* = 10, 9%),^49^^,^^58^^,^^68^^,^^74^^,^^78^^,^^87^^,^^89^^,^^92^^,^^99^^,^^113^ or those who could provide perspectives broader than their own experience (consumer advocates) (*n* = 2, 2%).^34^^,^^56^ Consumer motivation, education, and relevant skills were also considered (*n* = 3, 3%).^49^^,^^56^^,^^117^

#### Reporting Characteristics of Consumers

One hundred one articles (95%) gave details about the number of consumers involved (Supplementary Table S4), and of these 66 (62%) provided 1 or more characteristics of consumers they involved, including gender, disease stage, and ethnicity.^10^^,^^11^^,^^23^, ^24^, ^25^, ^26^, ^27^, ^28^, ^29^, ^30^, ^31^, ^32^, ^33^, ^34^, ^35^, ^36^, ^37^, ^38^^,^^40^, ^41^, ^42^, ^43^, ^44^, ^45^, ^46^, ^47^, ^48^^,^^51^^,^^52^^,^^56^, ^57^, ^58^, ^59^, ^60^, ^61^, ^62^, ^63^^,^^66^, ^67^, ^68^^,^^73^, ^74^, ^75^, ^76^, ^77^, ^78^^,^^82^^,^^83^^,^^87^, ^88^, ^89^, ^90^, ^91^, ^92^, ^93^^,^^95^^,^^98^^,^^99^^,^^103^, ^104^, ^105^, ^106^, ^107^^,^^111^^,^^112^^,^^115^^,^^118^^,^^121^

#### Representation and Inclusion

Although some authors described seeking diverse consumers based on age, employment status, and CKD stages or diagnoses,^30^^,^^37^^,^^44^^,^^45^^,^^47^^,^^49^ some indicated that minoritized ethnic groups (including Asian, African American, Indigenous Peoples) were difficult to reach and engage.^28^^,^^37^^,^^40^^,^^53^ Some researchers were conscious about the disease and treatment burden of CKD and involved more patients to allow for absences or periods of sickness (*n* = 2, 2%).^49^^,^^68^ The need for demographic “representation” among consumers was challenged, noting that this was not required for other members of the research team.^57^

#### Defining Consumer Roles

Consumers held various roles ranging from observing, informing, and engaging with researchers from their personal experience (e.g., information sessions, surveys, and focus groups) to driving research projects (e.g., patient led research network). The range of consumer roles with descriptions and examples of potential responsibilities are shown in Table 1.^23^, ^24^, ^25^, ^26^, ^27^, ^28^, ^29^, ^30^, ^31^, ^32^, ^33^, ^34^, ^35^, ^36^, ^37^, ^38^, ^39^, ^40^, ^41^, ^42^, ^43^, ^44^, ^45^, ^46^, ^47^, ^48^, ^49^, ^50^, ^51^, ^52^, ^53^, ^54^, ^55^, ^56^, ^57^, ^58^, ^59^, ^60^, ^61^, ^62^, ^63^, ^64^ Each category offers different degrees of involvement and can be adapted based on the patient’s capacity; for example, partial involvement can be both appropriate and meaningful. Patients might be able to provide input in a flexible manner or participate in specific aspects of the research process. Some consumers felt there was a lack of clarity about their roles and responsibilities—“there was no profile of what is expected of a lay coresearch, and no guidance on the activities I would need to take part in”^56^ and suggested the need for a structured job description or terms of reference.

#### Stages of the Research

Consumer involvement across the stages of research is shown in Table 2.

#### Involvement in Setting Priorities

Forty-seven articles (44%) included consumer involvement in setting research priorities,^23^, ^24^, ^25^, ^26^, ^27^, ^28^^,^^30^^,^^31^^,^^34^^,^^35^^,^^37^^,^^39^^,^^40^^,^^42^, ^43^, ^44^^,^^46^^,^^47^^,^^49^^,^^54^^,^^58^^,^^63^^,^^64^^,^^66^^,^^67^^,^^70^^,^^71^^,^^73^^,^^74^^,^^77^^,^^79^^,^^80^^,^^83^^,^^84^^,^^87^^,^^88^^,^^90^^,^^92^^,^^94^^,^^95^^,^^97^^,^^99^^,^^102^^,^^108^^,^^116^^,^^117^^,^^121^, ^122^, ^123^, with most using the James Lind Alliance—Priority Setting Partnership approach (*n* = 8).^26^, ^27^, ^28^^,^^37^^,^^44^^,^^45^^,^^80^^,^^82^ Priorities were elicited through involvement as members of the steering committees, surveys, and workshops.

#### Involvement in Research Design

Consumers contributed to protocols; advised on acceptability, feasibility, and practical aspects of interventions and study design; and provided input on recruitment and retention (*n* = 66, 62%),^10^^,^^11^^,^^23^, ^24^, ^25^^,^^33^^,^^36^^,^^38^^,^^39^^,^^41^^,^^44^, ^45^, ^46^, ^47^^,^^49^^,^^55^^,^^56^^,^^58^, ^59^, ^60^, ^61^^,^^63^^,^^64^^,^^69^, ^70^, ^71^, ^72^, ^73^, ^74^, ^75^, ^76^, ^77^^,^^79^^,^^80^^,^^83^^,^^84^^,^^86^, ^87^, ^88^^,^^90^, ^91^, ^92^, ^93^, ^94^, ^95^, ^96^, ^97^, ^98^, ^99^^,^^101^, ^102^, ^103^, ^104^, ^105^, ^106^, ^107^, ^108^^,^^110^^,^^112^^,^^114^, ^115^, ^116^, ^117^^,^^123^, ^124^, ^125^, ^126^ Consumers were primarily involved through advisory or reference groups, steering committees, or in workshops; and provided insights and ideas that led to changes and improvement in study or intervention design (e.g., preference for overnight dialysis rather than continuous wearable device,^36^ selection of outcomes and outcome measures [including core outcomes for trials],^38^^,^^41^^,^^49^ developed recruitment materials,^62^ and improved readability of materials^25^^,^^60^^,^^84^). Consumers helped to ensure that study design and materials were culturally safe and inclusive for minority groups.^25^^,^^46^^,^^58^^,^^59^^,^^63^^,^^83^

#### Involvement in Data Collection

Consumers contributed to the development of questionnaires and interview guides, and conducted interviews and focus groups, (*n* = 40, 38%).^10^^,^^49^^,^^56^^,^^58^^,^^60^, ^61^, ^62^, ^63^^,^^67^^,^^71^, ^72^, ^73^, ^74^, ^75^, ^76^, ^77^^,^^79^^,^^83^, ^84^, ^85^^,^^87^^,^^89^, ^90^, ^91^^,^^93^^,^^95^^,^^98^^,^^99^^,^^101^^,^^102^^,^^105^^,^^106^^,^^111^^,^^112^^,^^116^^,^^118^^,^^123^^,^^126^^,^^127^ They established rapport, whereby participants felt “no barriers in explaining their stories,”^34^ and patients identified ways to ensure consumers felt safe participating (e.g., having a native speaker or translator in the interview).^58^ Consumers identified strategies to address barriers to recruitment (e.g., provide a patient navigator or advocate).^63^

#### Involvement in Data Analysis

Thirty-nine articles (37%) described involving consumers in data analysis.^10^^,^^26^^,^^29^^,^^34^^,^^44^, ^45^, ^46^^,^^49^^,^^55^^,^^56^^,^^58^^,^^60^^,^^62^^,^^71^, ^72^, ^73^, ^74^^,^^76^^,^^79^^,^^84^^,^^86^, ^87^, ^88^^,^^90^^,^^91^^,^^93^^,^^95^^,^^98^^,^^99^^,^^101^^,^^102^^,^^105^^,^^106^^,^^111^^,^^112^^,^^116^^,^^118^^,^^123^^,^^126^ Consumers were involved in thematic analysis of qualitative studies, provided feedback on preliminary findings, worked with researchers to analyze the data, or led analyses.^29^^,^^34^^,^^44^^,^^45^^,^^55^^,^^56^^,^^58^^,^^62^ Authors noted that partnering with consumers in analysis yielded more nuanced findings and unique insights of the consumer’s context not captured by researchers, for example related to the burden of dialysis.^34^

#### Involvement in Disseminating Findings

Consumers presented findings at conferences, gave lectures at meetings and events, authored papers, produced lay summaries, developed educational materials (i.e., videos, brochures) and attended conferences as delegates (*n* = 47, 44%).^10^^,^^11^^,^^24^^,^^34^^,^^39^^,^^49^^,^^53^^,^^55^^,^^56^^,^^62^^,^^63^^,^^67^^,^^68^^,^^71^, ^72^, ^73^, ^74^, ^75^, ^76^, ^77^, ^78^, ^79^^,^^83^^,^^84^^,^^87^, ^88^, ^89^, ^90^^,^^94^^,^^95^^,^^98^^,^^99^^,^^101^, ^102^, ^103^^,^^105^^,^^112^, ^113^, ^114^^,^^116^^,^^118^^,^^119^^,^^123^^,^^126^^,^^128^ Fifty-six articles (53%) included consumer coauthors,^10^^,^^11^^,^^24^^,^^27^^,^^28^^,^^30^^,^^32^^,^^34^^,^^36^, ^37^, ^38^^,^^41^^,^^43^, ^44^, ^45^^,^^52^, ^53^, ^54^^,^^56^^,^^57^^,^^59^^,^^62^, ^63^, ^64^^,^^66^^,^^67^^,^^70^^,^^71^^,^^73^^,^^74^^,^^76^, ^77^, ^78^, ^79^^,^^85^, ^86^, ^87^, ^88^^,^^90^^,^^92^^,^^95^^,^^97^^,^^98^^,^^100^^,^^102^^,^^105^^,^^106^^,^^108^^,^^110^^,^^112^^,^^113^^,^^116^^,^^119^^,^^123^^,^^125^^,^^127^^,^^128^ including 12 consumer first authors.^11^^,^^52^^,^^53^^,^^56^^,^^88^^,^^98^^,^^99^^,^^114^^,^^119^^,^^120^^,^^124^^,^^126^ Twenty-two articles (21%) included an acknowledgment of consumers involved, without any consumer coauthors. ^25^^,^^29^^,^^33^^,^^40^^,^^42^^,^^47^^,^^49^^,^^61^^,^^69^^,^^72^^,^^80^^,^^82^^,^^83^^,^^89^^,^^91^^,^^93^^,^^96^^,^^104^^,^^107^^,^^111^^,^^117^^,^^118^

#### Involvement in the Implementation of Findings

Consumers implemented study interventions through pilot testing, providing feedback and identifying perceived barriers to the interventions (*n* = 37, 35%).^29^^,^^32^^,^^36^^,^^42^^,^^49^^,^^52^^,^^57^^,^^58^^,^^60^^,^^62^^,^^63^^,^^71^, ^72^, ^73^^,^^78^^,^^79^^,^^83^^,^^85^^,^^87^^,^^88^^,^^90^^,^^95^^,^^98^^,^^99^^,^^102^^,^^104^, ^105^, ^106^^,^^110^^,^^114^^,^^116^^,^^117^^,^^119^^,^^120^^,^^123^^,^^128^ One example consisted of consumer advisory council members modifying the implementation of an intervention to ensure it “reflected the unique culture and circumstances of the community.”^58^ Consumers were involved in knowledge translation activities (e.g., videos and educational materials), guiding implementation of a new model of care, enacting legislation (access to dialysis),^52^ streamlining regulatory decision-making processes (e.g., medical device approval with the US Food and Drug Administration), and consumers were also involved in prioritizing and selecting topics/outcomes for clinical guidelines (*n* = 9, 8%).^1^^,^^31^^,^^32^^,^^35^^,^^37^^,^^41^^,^^42^^,^^44^^,^^47^

#### Involvement in Evaluation

Twenty-one articles (20%) involved consumers in evaluating the project or intervention.^11^^,^^24^^,^^25^^,^^29^^,^^36^^,^^44^^,^^46^, ^47^, ^48^, ^49^^,^^58^^,^^59^^,^^61^^,^^73^^,^^77^^,^^78^^,^^86^^,^^98^^,^^100^^,^^103^^,^^105^^,^^123^ Examples of methods included informal discussions,^36^ workshops or meetings^46^^,^^49^^,^^58^^,^^61^^,^^77^ (e.g., identified consumer educational needs or interests and meaningful intervention for First Nations people), consumer testimonials^36^ (e.g., increased machine portability, automatic transmission of treatment data, and remote monitoring, surveys or written feedback^24^^,^^25^^,^^36^^,^^47^^,^^48^^,^^59^^,^^122^ (e.g., need for simpler language), and interview studies^29^^,^^44^ (e.g., preference for in-person over online participation). Consumers also communicated the impact of the project back to participants and the broader community using plain language and promoted future projects.

#### Resources

Several articles outlined the necessary resources for the involvement of consumers, detailing financial support and remuneration to support their engagement in research, along with the human resources needed to provide education, training, and logistical support.^67^^,^^76^^,^^80^^,^^89^^,^^90^^,^^92^^,^^93^^,^^105^^,^^110^ In efforts to mitigate risks, 1 article discussed developing an insurance plan for physical or financial losses resulting from study participation, which was developed by their patient-led leadership committees.^110^

#### Financial Support

Twenty-one articles (20%) mentioned financial support for consumers.^11^^,^^25^^,^^36^^,^^40^^,^^41^^,^^46^^,^^47^^,^^49^^,^^56^^,^^58^^,^^67^^,^^76^^,^^80^^,^^82^^,^^89^^,^^90^^,^^92^^,^^93^^,^^101^^,^^105^^,^^110^ This included travel grants to consumers to attend workshops,^36^ reimbursements for travel,^11^^,^^15^^,^^40^^,^^41^^,^^47^^,^^56^^,^^58^^,^^82^ and/or a stipend or honorarium.^11^^,^^25^^,^^46^^,^^49^^,^^56^ Four articles included budgets for consumer involvement,^56^^,^^80^^,^^101^^,^^110^ and 1 reported specific funding for a consumer coresearcher.^56^ It was recommended that consumers be reimbursed for expenses incurred (time off work, transport, accommodation, and childcare costs)^84^ and providing compensation would allow for more diverse involvement.^57^

#### Education or Training

Training included formal workshops (for consumers, health practitioners, and researchers),^11^^,^^27^^,^^33^^,^^58^^,^^62^^,^^67^^,^^71^^,^^74^^,^^80^^,^^88^^,^^94^^,^^95^^,^^99^ informal discussion-based learning/feedback tailored to specific tasks,^24^^,^^56^ and learning-by-doing approaches,^34^^,^^56^ and focused primarily on research methods and subject matter content. Other support provided for consumers include induction or orientation programs, mentoring, peer-to-peer support, and liaisons who provided consumers with practical, logistical, and technical support (e.g., accessing videoconferencing and supporting mobility needs).^49^^,^^62^ Providing education or training was found to enable consumers to make more meaningful contributions.^10^^,^^36^^,^^57^

#### Evaluation of Impact

Twenty-four publications (23%) provided an evaluation report of the consumer involvement activities, although most were informal and brief.^11^^,^^24^^,^^31^^,^^33^^,^^34^^,^^37^^,^^44^^,^^45^^,^^47^^,^^49^^,^^56^^,^^58^, ^59^, ^60^^,^^62^^,^^68^^,^^76^, ^77^, ^78^^,^^100^^,^^101^^,^^105^^,^^119^^,^^122^ The benefits, challenges and long-term impacts from the researcher and consumer perspectives are provided in Table 3. The principles and strategies identified within these articles as leading to beneficial and meaningful involvement are shown in Table 4.

**Table 3:** Challenges, benefits, and impacts

Evaluation component	Examples from included papers	
	Predominantly affecting consumers	Predominantly affecting researchers
Benefits to the research project	Improved content and clarity of information^59^^,^^101^ Improved communication (type, language, format and timing of information)^11^^,^^24^^,^^33^ Encouraged adherence to timelines^62^ Improved knowledge translation activities (e.g., educational videos, lay summaries)^45^^,^^47^^,^^62^ More useful outputs for end users (clinicians, consumers)^47^^,^^101^ Increased agreement on priorities between stakeholders^122^ Cultural safety, sensitivity^58^	Increased recruitment rates^101^^,^^122^ Increased retention/adherence rates^60^^,^^101^ Improved relevance/importance of topics, interventions^24^^,^^31^^,^^47^^,^^59^ Validation of findings^101^ Richer data collection (interviews)^34^^,^^62^ Captured research findings missed by researchers (questions, themes, topics, nuances)^31^^,^^34^^,^^37^^,^^62^
Challenges /barriers	Lack of shared language (lay versus scientific/medical jargon)^49^ Limited research training^45^^,^^49^ Timing/scheduling, need for flexibility and time commitment^45^^,^^49^ Transparency around contributions/impact^49^ Navigating different timeline expectations, time between meetings, adherence to timelines, time to implementation^56^^,^^62^	Navigating dynamics between researchers and consumers (including consumer partners and consumer participants)^33^^,^^34^ Understanding ethics requirements for including consumers as partners in research^49^^,^^62^ Resistance of researchers toward questions generated by nonscientists^49^ Involving broad/diverse consumers (“engaged predisposition”)^45^ Documenting/recording PPI activities^56^
Long term impacts	Enhanced patient advocacy (consumers representatives, researchers, clinicians)^45^^,^^56^ Continuity of involvement in future projects^45^ More empathy/compassion for and understanding of one another (consumers and researchers/clinicians)^11^^,^^33^^,^^45^^,^^58^^,^^62^ Sense of empowerment/meaning for consumers^49^ Support/perspective/community derived from meeting other consumers^45^ Enhanced knowledge and understanding for consumers (disease progression, symptoms, treatment options, general kidney health, research process)^11^^,^^45^ Confidence for consumers to advocate themselves in clinical settings^11^^,^^45^	Shift in research interests/focus to be more patient-centred^45^ Enhanced knowledge and understanding for researchers (patient-oriented research, how to involve consumers, value of involvement)^11^^,^^45^ New/modified clinical approaches (e.g., including consumer priorities in clinic questionnaire, guidelines)^31^^,^^45^^,^^47^ Connection between the research team and the broader community^58^ Patient Reported Outcome sub study development^60^

**Table 4:** Strategies and principles for successful involvement

Principle	Strategies
Avoiding tokenism	Build ongoing, trusting and authentic relationships^57^^,^^63^^,^^64^^,^^84^ Encourage open dialogue throughout^34^^,^^49^^,^^53^^,^^84^ Engage in meaningful activities^53^ (e.g., involve consumers early and in upstream decision-making with the rest of the research team)
Respecting consumers’ time and capacity	Clarify roles, responsibilities and deliverables (include in study protocol)^53^^,^^57^^,^^63^^,^^84^ Develop a Terms of Reference/ Memorandum of Understanding^11^^,^^58^^,^^84^ Minimize burden on consumers (time, financial)^35^^,^^53^^,^^56^, ^57^, ^58^ Schedule activities on days/times that enable attendance^24^^,^^56^^,^^58^ Schedule activities in locations accessible to consumers (or consider access e.g., university/hospital buildings)^11^ Elicit preferences for communication modalities—offer multiple modes of attendance/participation, where possible allow for face-to-face meetings^11^^,^^24^^,^^44^^,^^57^
Valuing consumer knowledge and expertise	Provide regular project updates/results^57^ Demonstrate impact of involvement^36^^,^^44^^,^^49^ (e.g., provide written feedback to consumers to show how their contributions impacted the research) Build capacity (e.g., training, peer mentoring)^35^^,^^57^ Reimbursement for time and expenses^11^^,^^57^^,^^84^
Consideration for the patient journey	Be sensitive to patient wellness/treatment stage^57^^,^^60^ Allow for varied degrees of involvement according consumer preferences^49^ Involve more than “needed” with the understanding that attendance may fluctuate (“over-recruit”)^49^^,^^60^ Involve consumers in determining timelines^58^
Considering the context of the “whole” person	Understand their skills and interests, aside from being a “patient” or “carer”^63^ Work within their availability—consider existing work, personal and family/social commitments^53^^,^^56^^,^^57^ Accommodate language/learning needs^49^ (e.g., provide translator; conduct in own language; ensure appropriate communication for level of education) Respect diversity and differences^34^ “Remember the humanity of the person”^53^ (e.g., remember that being a patient is only one aspect of a person’s life and they will have competing priorities including family, career, social)
Sharing of power and providing support	Provide orientation/induction^84^ Buddy/liaison role^49^^,^^60^^,^^84^ Provide practical support where needed (e.g., technical—videoconferencing, transport)^56^ Involve consumers in decisions regarding training desired/required^11^^,^^49^ Use plain language^36^^,^^57^ Training for researchers^57^ Provide “safe” environments to empower contributions^34^ (e.g., ensure proper consent and confidentiality; establish relationships with community leaders) Involve more than one consumer in any group^34^^,^^42^^,^^60^
Developing consumer networks/systems to support continued involvement	Establish a board/panel/council of consumers who are ready to become involved^49^^,^^57^ Identify opportunities for future involvement^63^ Cultivate partnerships/networks with patient advocacy organizations and health care professionals^36^^,^^49^^,^^57^ Explore opportunities for web-based platforms/networks^57^
Reciprocity	Facilitate bidirectional knowledge exchange and translation^43^^,^^57^ Provide access to education/training/tools^36^ Empower health ownership^57^ (e.g., provide education/training about disease management)
Inclusion of perspectives	Involve diverse consumers^64^ Where possible and relevant, look to include vulnerable and minority groups^49^
Valuing the local	Engage local community members as “cultural brokers”^35^^,^^43^ (e.g., employ a community leader/member as a coresearcher and support them to liaise between the community and the research team)
Cultural safety and competence	Understand the distinct needs and expectations of community groups^35^ Acknowledge importance of spiritual beliefs^58^ (e.g., engage with the community to understand how their beliefs can be acknowledged and accommodated) Provide regular updates to and seek feedback from the community, include contact information and photographs of the research team^58^ Establish ground rules for confidentiality^11^

## Discussion

There has been an exponential increase in articles that describe consumer involvement in research in CKD with more than 68% published in the past 5 years. Greater levels of consumer involvement in recent years is apparent, where consumers are positioned in more meaningful roles to impact the design and conduct of the research (Figure 2). Those involved at higher levels with greater decision-making power (e.g., coresearcher or patient partner) were often highly educated, with some medical training or academic experience. Patients in disadvantaged and vulnerable circumstances were less involved in research, and they were often recruited through established research networks and professional societies, their hospitals or clinics, and/or registries and databases.

Consumer involvement is occurring, increasing, and is feasible. Throughout the research stages, consumers were involved in diverse roles and engaged in various activities. Authors described diverse methods for identifying potential consumer partners and pathways to involving them in research. In addition, some articles describe patient-driven projects or comprise patient-oriented research networks (Supplementary Table S4). Some articles described patient or caregiver involvement in identifying interventions and outcomes for trials and observational studies.

Across the articles, the authors describe principles and strategies employed to maximize benefits and mitigate challenges; and outline the financial, logistical, training, and personnel resources needed to support consumers (Table 4). Many patients with CKD, particularly those receiving dialysis or at end-of-life experience fatigue, cognitive impairment, and we acknowledge that the fluctuating health in patients with CKD may be barriers to involvement across all stages of research. In addition, historical power imbalances and past negative experiences with the health care system may lead to skepticism about whether their contributions will be valued or acted upon.^57^^,^^63^^,^^64^^,^^84^ For patients in particular, the burden of self-management responsibilities and time-consuming and invasive life-sustaining therapy (i.e., dialysis), and prolonged periods of illness, including symptoms of fatigue and impaired cognitive function, might make involvement exceptionally challenging; whereas for caregivers, the burden of caring responsibilities can become all-consuming and may lead to burnout.^129^ In these cases, partial involvement may be both practical and meaningful. Articles expressed optimizing support structures achieved through making adaptions and improvements to existing resources, and development of new ones when feasible, to address identified gaps.^79^ The reported impacts of these consumer involvement initiatives included improved communication, increased recruitment, retention and adherence, richer data collection, validation of findings, improved knowledge translation, more useful outputs for end-users and increased agreement on priorities between stakeholders.

Differences in consumer involvement were observed across different research stages, particularly in terms of activities and the number of consumers involved. The majority of consumers were involved in single large group activities (e.g., workshops and surveys) in the preparation phases (i.e., priority settings and design) with limited opportunity to provide feedback or ensure that their voices have been carried through to the execution and translation phases, which has been echoed in other reviews.^4^^,^^16^^,^^130^ Although consumer involvement in data collection and analysis in qualitative research yielded better quality data,^4^ in these stages fewer consumers were involved, and this was typically through a coinvestigator or patient-partner role or as members of a consumer advisory group.

As shown by this review, there are opportunities to strengthen consumer involvement based on evidence from published articles, including identifying and selecting consumers; setting expectations; support and capacity building; and evaluating processes and impact (including consistent reporting and publishing of involvement). Inducting consumers formally, providing role clarity, flexible schedules, resolving any conflicts promptly, and offering financial or logistical support are key to successful consumer involvement.^13^^,^^14^^,^^109^^,^^131^ The strategies and principles for consumer involvement in research are outlined in Table 4.

We conducted a comprehensive search of articles on consumer involvement in published CKD research and a descriptive synthesis according to an explicit framework; however, there are some potential limitations. First, we excluded articles that were not published in English because of insufficient resources for translation. There is inconsistent and variable reporting of consumer involvement in publications in nephrology. Transparency of consumer involvement would be improved with clearer and consistent “Patient and Public Involvement” statements and formal acknowledgments of consumer coauthorship, where applicable as seen in the BMJ group of journals, where it is a requirement of every article submitted.^132^

The evaluation of consumer involvement processes and the impact of their involvement was limited across selected articles. We suggest that further efforts are needed in future research to evaluate the process and impact of consumer involvement (including impact on recruitment and adherence, and community knowledge or awareness). However, it is difficult to develop best practice for consumer involvement while reporting and publishing remains scant. Although the GRIPP/GRIPP2^19^^,^^20^ checklists have been developed to improve reporting, only 4 articles (4%) used this guidance in their reporting.^56^^,^^62^^,^^76^^,^^77^ The GRIPP2 checklist offers a structured approach to reporting patient and public involvement in research and includes various aspects, such as recruitment, design, and impact measurement.^19^^,^^20^ It may not be possible to include all the reporting items from GRIPP2 in a manuscript; however, these details could be included in supplementary files or warrant a separate publication (Supplementary Table S5). More is required to strongly encourage or mandate the reporting of consumer involvement in published journals. The INVOLVE toolkit, the Patient-Centered Outcomes Research Institute Engagement Rubric and their “Science of Engagement” program, the NHMRC statement on Consumer and Community Involvement in Health and Medical Research, and the National Framework for Consumer Involvement in Cancer Control are freely accessible and designed resources to support the early involvement of consumers, provide clarity of roles and responsibilities, provide access to resources, training, and education.^3^^,^^18^^,^^133^^,^^134^ Alternatively, existing frameworks for individual study types (e.g., CONSORT and STROBE) could be amended to include important items relating to consumer involvement in a table or figure or require justification for not involving consumers. By adhering to these guidelines, researchers can provide greater transparency and ensure their publications effectively reflect the contributions of consumer involvement.

The included articles in this review demonstrate that involving consumers in all aspects of CKD research is possible and beneficial. Future research could prioritize incorporating diverse perspectives, supporting patients’ health needs, developing organizational processes, financially and logistically supporting consumers, and enabling their ongoing meaningful involvement with greater decision-making power. Research teams should recognize the nuances of the different levels of involvement in research as this can help ensure that consumer involvement is appropriate and tailored to the context and is respectful of the patients' capacities and preferences. To enhance the impact of consumer involvement in CKD research, researchers could adopt participatory research methodologies such as action research or design-based research, which emphasize continuous collaboration and iterative feedback.^9^^,^^25^^,^^135^, ^136^, ^137^ These approaches actively involve patients and caregivers in shaping research questions, methodologies, and interventions, ensuring that studies remain relevant and responsive to their needs. These strategies may offer actionable guidance for researchers to enhance the depth and authenticity of patient engagement. To bolster best practice in CKD research, it is essential to conduct formal and published evaluations of consumer involvement processes and their impacts. Future studies should adopt more comprehensive reporting frameworks, such as the GRIPP2 checklist. In addition, the kidney research community should proactively adopt evolving best practices for the inclusion of consumers, and by doing so, they can align with the advancements seen in other health disciplines as they develop. Embracing this evolving science of best practices for the inclusion of consumers ensures that the contribution of consumers are effectively integrated and valued.

## Disclosure

DC is supported by the 10.13039/501100000925National Health and Medical Research Council (NHMRC) Center of Research Excellence Grant (2007026). TG is a recipient of the NHMRC postgraduate Scholarship (1169149). AJ is a recipient of the NHMRC postgraduate Career Development Fellowship (APP1106716). AKV receives grant support from a Queensland Advancing Clinical Research Fellowship and an NHMRC Emerging Leader Grant (1196033). DT is a recipient of a NHMRC Emerging Leadership Grant (1197337). All the other authors declared no competing interests.

## Supplementary Material

Supplementary File (PDF) **Table S1.** Search strategy. **Table S2.** Gray literature search. **Table S3.** Detailed characteristics of included articles. **Table S4.** Definitions of terms relating to consumer involvement. **Table S5.** Guidance for reporting involvement of patients and public 2: short form checklist. PRIMSA checklist.

## Footnotes

Supplementary File (PDF)
**Table S1.** Search strategy. **Table S2.** Gray literature search. **Table S3.** Detailed characteristics of included articles. **Table S4.** Definitions of terms relating to consumer involvement. **Table S5.** Guidance for reporting involvement of patients and public 2: short form checklist. PRIMSA checklist.
