# Supplementary material for: Systematic Review of Patient and Caregiver Involvement in CKD Research
Source: Kidney Int Rep. 2025 Mar 17;10(6):1657–72. doi: 10.1016/j.ekir.2025.03.018 (PMC12232971; doi:10.1016/j.ekir.2025.03.018)
Supplement: Supplementary File (PDF) — Table S1. Search strategy. Table S2. Gray literature search. Table S3. Detailed characteristics of included articles. Table S4. Definitions of terms relating to consumer involvement. Table S5. Guidance for reporting involvement of patients and public 2: short form checklist. PRIMSA checklist. [file mmc1.pdf]

TABLES: Supporting documentation for systematic review.

Supplementary Table 1. Search strategy.

EMBASE 1947 to 18th August 2023

#	Searches	Results
1	exp Kidney Failure, Chronic/	150156
2	Chronic kidney disease.mp or exp Renal Insufficiency, chronic/	222455
3	exp Peritoneal Dialysis/ or exp Dialysis/ or exp Renal Dialysis/ or exp Peritoneal Dialysis, Continuous Ambulatory/ or dialysis.mp	293372
4	hemodialysis/	135637
5	exp Nephrology/	23482
6	exp Kidney Transplantation or kidney transplant.mp	184316
**7**	**1 or 2 or 3 or 4 or 5 or 6**	**603915**
8	consumer participation/	57881
9	Patient participation/	35540
10	8 or 9	92968
11	(patient* or public or lay or people or consumer* or user* or citizen or parent or parents or child*).ti	4649213
12	(paticipat* or involve* or engag* or consult* or collaborat* or conducting or conducted or contrib*).ti	487224
13	(questionnaire* or interview* or focus group* or workshop*or peer led or research* or self-report* or qualitative or patient led or public led or self-rating or self rated or development).ti	1113363

14	11 and 12 and 13	4993
15	((health or research) and (partners or partnership)).ti,ab	76331
**16**	**10 or 14 or 15**	**171204**
**17**	**7 and 16**	**1379**

Ovid Medline ® 1946 to 18th August 2023

#	Searches	Results
1	exp Kidney Failure, Chronic/	101094
2	Chronic kidney disease.mp or exp Renal Insufficiency, chronic/	171912
3	exp Peritoneal Dialysis/ or exp Dialysis/ or exp Renal Dialysis/ or exp Peritoneal Dialysis, Continuous Ambulatory/ or dialysis.mp	200420
4	hemodialysis/	102223
5	exp Kidney Transplantation/ or kidney transplantation.mp	112845
**6**	**1 or 2 or 3 or 4 or 5**	**388105**
7	consumer participation/	18474
8	Patient participation/	29467
9	7 or 8	47512
10	(patient* or public or lay or people or consumer* or user* or citizen or parent or parents or child*).ti	3427082
11	(paticipat* or involve* or engag* or consult* or collaborat* or conducting or conducted or contrib*).ti	439779
12	(questionnaire* or interview* or focus group* or workshop* or peer led or research* or self-report* or	971534

	qualitative or patient led or public led or self rating or self rated or development).ti	
**13**	**10 and 11 and 12**	**4252**
14	((health or research) and (partners or partnership)).ti,ab	56049
**15**	**9 or 13 or 14**	**104970**
**16**	**6 and 15**	**820**
17		

PsycINFO 1806 to 18th August 2023

#	Searches	Results
1	exp Dialysis/ or exp Kidney Diseases or exp Hemodialysis/ or Kidney Failure.mp	4346
2	Chronic kidney disease.mp. or exp hemodialysis/	2490
3	Renal failure.mp.	1158
4	Nephrology.mp	329
5	Kidney transplant.mp. or exp Organ Transplantation/	5442
**6**	**1 or 2 or 3 or 4 or 5**	**10407**
7	consumer participation/	0
8	Patient participation/	3178
9	(patient* or public or lay or people or consumer* or user* or citizen or parent or parents or child*).ti	723253
10	(paticipat* or involve* or engag* or consult* or collaborat* or conducting or conducted or contrib*).ti	120418
11	(questionnaire* or interview* or focus group* or workshop*or peer led or research* or self-report* or	340398

	qualitative or patient led or public led or self rating or self rated or development).ti	
12	9 and 10 and 11	2186
13	((health or research) and (partners or partnership)).ti,ab	35372
**14**	**8 or 12 or 13**	**40289**
**15**	**6 and 14**	**89**

Supplementary Table 2: Grey Literature Search

Journals

1. Research Involvement and Engagement (n=15 screened, n=2 unique included)
2. The Patient (n=82 screened, n=0 unique included)
3. Health Expectations (n=5 screened, n=0 unique included)

Website search

1. INVOLVE - https://www.invo.org.uk/ (n=6 screened, n=0 unique included)
2. International Society of Nephrology - https://www.theisn.org/ (n=1 screened, n=1 unique included)
3. Kidney Diseases Improving Global Outcomes - https://kdigo.org/ (n=1 screened, n=0 unique included)
4. Can-SOLVE CKD Network - https://www.cansolveckd.ca/ (n=1 screened, n=0 unique included)
5. American Society of Nephrology - https://www.asn-online.org/ (n=0 screened, n=0 unique included)
6. Kidney Research UK - https://kidneyresearchuk.org/ (n=3 screened, n=0 unique included)
7. Kidney Health Australia - https://kidney.org.au/ (n=6 screened, n=3 unique included)

Citing GRIPP/GRIPP2

n=6 screened

n=4 unique included

Supplementary Table 3. Detailed characteristics of included articles.

Author	Activity	N (%) *	Country	Concept	Article informed a clinical trial	Article informed clinical guidelines
**Aguilar-Gonzalez (2022)**	Co-researchers.	62 (95%)	GUAT	Developed several educational activities to highlight the importance of kidney health and adequate fluid intake.		
**Archdeacon (2013)**	Board of directors (voting)<br>Patient advocacy groups	2 (10)<br>^	US	KHI, improve safety, foster innovation.		
**Banerjee (2020)**	Survey development.	NA	NA	Consulted in the development of online survey.		
**Barnieh (2014)**	Steering Committee<br>Online Survey<br>Workshop	5 (45%)<br>^<br>16 (53%)	CAN	Research priorities for patients on or nearing dialysis.	Yes	
**Beaubien-Souligny (2022)**	Patient partners	5 (63%)	CAN	Provide insight and feedback at specific moments in the research.		
**Bernstein (2019)**	NA	NA	CAN, US, UK	Patient involvement in nephrology nursing research.		
**Bishop (2017)**	Patient Involvement group with team lead	^	UK	Acceptability of exercise for transplant recipients.	Yes	
**Blackburn (2018)**	5/200 projects reported involvement with renal-urogenital patients	NA	UK	Extent, quality, and impact of patient and public involvement in primary care research.		
**Blomqvist (2010)**	Co-researching group	4 (100)	SE	Supporting daily life of patients with CKD (in clinic and outside).		

**Bonventure (2019)**	Patient Advisory Committee<br>Patient Advisory Group<br>National Kidney Foundation	8(100)<br>3 (100)<br>^	US	KHI Technology Roadmap for innovative approaches to KRT.		
**Browne (2020)**	Patient partners	10	US	Provide insight and feedback at specific moments in the research.		
**Burr (2022)**	Steering committee members	^	UK	Attendance in steering committee meetings, providing commentary on research materials, and involvement in dissemination activities.	Yes	
**Carter (2022)**	Workshops	50 (36%)	AUS, CAN, US	Attend one or both workshops, provide feedback on preworkshop materials and draft report.	Yes	
**Cho (2019)**	Steering Committee<br>Focus Group	3<br>154 (100)	AUS, KR, FR	Identification and prioritization of core outcomes for research in ADPKD.		Yes
**Clemens (2019)**	Patient research partners	2 (29%)	UK, CAN	Patient-centered care for diabetes and advanced CKD.		
**Conway (2019)**	American Association of Kidney Patients (AAKP)	NA	US	Integrating patient input into kidney disease research - examples from AAKP.		
**Cukor (2016)**	Patient advisory groups/councils Patient advocacy/research groups Steering committee members	NA	US	Patient engagement in PCORI funded studies in kidney disease.		
**Curtis (2021a)**	Patient Partners	10	CAN	Recruitment, educational materials and advisory on knowledge translation strategies.		

**Curtis (2021b)**	Patient partners	^	CAN	overseeing the execution of knowledge translation activities including, but not limited to, community selection criteria, community engagement procedures, and review of communication materials and strategies.	
**Demian (2017)**	Consumer co-researcher/author	1 (20)	CAN	Opportunities for engaging patients in kidney disease research.	
**Donald (2019)**	Patient partners	17(71%)	CAN	Involved in the study design, participated in data collection, reviewed final outputs, and contributed to manuscript preparation and dissemination.	
**Dorough (2021)**	Interviews, Consumer Advisors	80 (76%)	US	Education materials development, interviews.	
**Doyle (2019)**	Focus group<br>Patient information meeting<br>Patient representative group	8 (100)<br>65 (100)<br>^	IE	Consumers involved in design of intervention - focus group, meeting/workshop.	
**Duff (2018)**	Focus groups	^	AUS	Engaging Indigenous peoples in guideline development.	Yes
**Duncanson (2020)**	Consumer advisory board<br>Consumer co-researchers/authors<br>Consumer conference attendees:<br>in person<br>online	40(100)<br>2 (14)<br><br>40 (^)<br>383 (^)		Partnering with consumers in conferences.	

**Edwards (2022)**	Consumer Co-researcher	1(50)	AUS	Manuscript preparation and publishing.		
**Elliot (2018a)**	Consumer co-researcher<br>Interview participants (previously involved in steering committee, workshop and/or online Wiki platform)	1 (14)<br>12(52)	CAN	Long term views on JLA priority setting partnership for CKD research priorities.		Yes
**Elliot (2018b)**				Perceived significance of engagement (Patients, caregivers, HCPs) in research PSP – impact on participants.		
**Elliot (2021)**	Patient partners	N/A	CAN	Establish a platform for knowledge translation within and across provinces to facilitate the dissemination and implementation of research evidence.		
**Elliot (2023)**	Patient partners	24 (50%)	CAN	Identify themes with subthemes that characterized the dynamic nature of patient engagement and how participants integrated patients across the network initiatives.		
**Engels (2022)**	Steering group members<br>Focus groups<br>Surveys	3 (19%)<br>8 (50%)<br>125 (74%)	NL	Determined the target audience, scope, purpose, and general format of the patient decision aid.		
**Evangelidis (2017)**	Patient partners	N/A		Patients / Caregivers joined the Delphi Panel		Yes
**Farragher (2019)**	Key informants, patient partner, patient engagement research	^	CAN	Designed intervention of study, providing consultation and feedback, interpreting results and	Yes	

				strategies to optimize dissemination and uptake.
**Finderup (2019)**	Interviews<br>Advisory Board	1 (14)<br>2 (25%)	DK	Engaging patients in evaluating a shared decision-making intervention for dialysis choice.
**Finlay (2020)**	Patient partners	25 (76%)	CAN	Conduct a qualitative descriptive study using semi-structured interviews and content analysis.
**Fowler (2017)**	KHI Patient and Family Partnership Council	≤10 (100)	US	Strategic guidance to KHI – including patients, families, caregivers in KHI activities.
**Fowler (2022)**	Patient partners	25 (76%)	CAN	Conducted a qualitative descriptive study using semi-structured interviews and content analysis.
**Getchell (2020)**	Patient partners	N/A	CAN	Stakeholder consultation throughout research
**Getchell (2022)**	Patient partners, Steering committee members, advocates	4<br>^<br>^	CAN	Created resources, enhancing knowledge translation.
**Getchell (2023)**	Patient Partner	NA	CAN	Feedback / reviewing final manuscript.
**Guha (2020a)**	Commissioner	1(100%)	AUS	Manuscript preparation and publishing.
**Guha (2020b)**	Consumer co-researcher, Workshops	3 (10)<br>24(100%)	AUS	Research priorities for patient navigator programs for patients with CKD.

**Gutman (2020a)**	Consumer co-researchers<br>Workshop co-investigators	3 (18)<br>105(71)	AUS	Principles and strategies for involving patients and their families in CKD research.	
**Gutman (2020b)**	semi-structured interviews	14(48%)	CAN, US, AUS	Attended interviews.	
**Gutman (2022)**	Patient partners<br>Interview participants	1(7)<br>23 (100%)	AUS, US, DK, UK	Attended interviews, sharing their experience and attitudes with their involvement in research.	
**Hurst (2017)**	Project workgroup<br>Workshop	3 (21)<br>60 (55)	US	Involving consumers in the development of medical devices - KHI	
**Husbands (2019)**	Patient advisory group workshop	^	UK	To obtain feedback on the clarity and presentation of patient information sheets and questionnaires. Members of the PAG were given a summary of the proposed RCT at the beginning of the workshop.	Yes
**International Society of Nephrology**	NA	NA	INT	Guidance on how to involve consumers throughout the research process, particularly for clinical trials.	
**Isautier (2022)**	Patient Partner	1 (13)	AUS	Part of the development team and provided valuable feedback regarding app content and usability.	Yes
**Jamieson (2020)**	Co-researcher<br>Patient Advisory Groups<br>Interviews	1 (^)<br>^ (^)<br>102 (100%)	AUS	Interviews focused on Co-ordinating around patient commitments, general health and wellbeing, and medical treatment.	

**Kayler (2021)**	Surveys and Interviews	25 (100%)	US	Involved with development of surveys and interviews.	
**Kayler (2022)**	Consumer Advisory Board	5 (100%)	US	Assisted with substantiating providers' impressions of patient experiences.	
**Kayler (2023)**	Interviews<br>Steering Committee<br>Consumer Advisory Board<br>Patient Partners	31 (100%)<br>4 (^)<br>7 (9)<br>4(8)	US	Reflect the decision-making needs of diverse kidney failure patients and family members in our community.	
**Kelly (2019)**	SA Aboriginal Community Reference Group<br>Workshop Participants	3 (100)<br><br>19 (100)	AUS	Indigenous community involvement, guideline development, priority setting/scoping	
**Kerklaan (2020)**	Interviews<br>Steering Committee	30 (100%)<br>1 (^)	AUS	Directly involved in the study as participants in the interviews and a consumer co-researcher who was involved in the planning and design of the study.	
**Kirkham (2019)**	Indigenous Consumer Reference Group<br>Co-researcher	6 (100)<br>3 (12)	AUS	Enabled Indigenous knowledge to guide the project, indigenous priorities to be identified in this context and timely feedback of information to inform the strengths and priorities of the health service.	
**Knight (2016)**	Steering group<br>Patient organizations<br>Initial survey<br>Prioritisation survey<br>Workshop	^<br>^<br>113 (62)<br>117 (46)<br>11 (55)	UK	Defining shared priorities for future research in Kidney Transplantation	Yes

**Levin (2018)**	Steering Committee: 2012-2013<br>- 2014<br>Survey: 2012-2013<br>- 2014<br>Workshops: 2012-2013<br>- 2014<br>Patient co-researchers (training)	5 (50)<br>6 (60)<br>210 (100)<br>309 (100)<br>16 (47)<br>18 (72)<br>56	CAN	Canadians Seeking Solutions and Innovations to Overcome Chronic Kidney Disease (Can-SOLVE CKD): Form and Function – accelerating knowledge translation to clinical research and practice, top 10 research priorities for CKD.	Yes
**Logan (2023)**	Interviews<br>Focus Groups<br>Consumer Advisory Group<br>Co-Researchers	^	AUS	Reviewing trial participant materials for comprehensive assessments to increase attainment of patient-identified goals.	
**Lopez-Vargas (2019)**	Workshop	35 (47)	AUS	Research priority setting for childhood chronic conditions (including kidney disease).	
**Loud (2013)**	Consumer co-researcher<br>Advisory group	1 (33)<br>6 (100)	UK	Involving a consumer advisory group in improving variation in primary for patients with CKD (Quality improvement project).	
**Mader (2018)**	Patient led research network	^	UK	Patient led research hub.	
**Manera (2019)**	Steering committee<br>Focus groups: 126<br>Survey participants	2 (14)<br>126 (100)<br>207 (24)	INT	Developing a core outcome set for patients receiving peritoneal dialysis.	
**Manns (2014)**	Steering committee<br>Survey respondents<br>Workshop	5 (45)<br>210 (66)<br>16 (47)	CAN	Research priorities for patients on/nearing dialysis.	Yes
**Markossian (2021)**	Focus Groups/ Interviews<br>Stakeholder team members.	13 (100%)<br>5 (^)	US	Identified and discussed a list of requirements and preferences regarding the content, features, and	

				technical aspects of the mobile app to support self-management of chronic kidney disease.	
**Marks (2018)**	Co-researcher<br>Public Involvement<br>Research group<br>Reference group<br>Advisory group	1 (20)<br>15 (100)<br>4 (57)<br>4 (36)	UK	Roles of the co-researcher as part of the research team	
**Mc Laughlin (2020)**	Workshops<br>Steering Committee	22 (20%)<br>^	UK	Identifying integrated health services and social care research priorities in kidney disease in Wales through workshops.	
**Mick-Ramsay (2019)**	Co-researcher<br>Consultation participants<br>Top End Renal Patient Advisory & Advocacy Committee, KHA	1 (11)<br>^<br>^	AUS	Indigenous community involvement, guideline development, priority setting/scoping.	
**Miller (2017)**	Workshop	11 (100)	AUS	Priority topics/ outcomes for infectious diseases in HD patients.	Yes
**Molnar (2017)**	Patient advisory committees	NA	CAN	Opportunities and challenges for patient engagement in kidney research.	
**Moore (2020)**	Interview<br>Advisors	220 (100%)<br>^	UK	Provided invaluable comments on the initial draft of the stating dialysis questionnaires' (SDQ) and involved in interviews.	
**Murdoch (2021)**	Workshop<br>Patient Partners	11 (33%)<br>6 (15%)	CAN	Participate in the workshop. Provided feedback and assisted with preparation of manuscript.	
**Muscat (2021)**	Patient Co-Researcher	1(12)	AUS	Co-authorship involving with preparing manuscript and publishing.	

**Muthuramalingam (2020)**	Commissioner, Patient research partner	5 (6)	AUS	Lead authorship involving with preparing manuscript and publishing.	
**Natale (2020)**	Workshop	105 (71%)	AUS	Involved in workshop identifying themes for improving recruitment and retention in clinical trials.	
**Natale (2023)**	Co-researcher	1 (^)	AUS	Manuscript (Validation and Visualization) and publishing (original draft and review/editing).	
**Navaneethan (2015)**	NA	NA	US	Patients as stakeholders in setting priorities for kidney disease research.	
**Nicholls (2021)**	Interviews<br>Focus Groups	5(100%)<br>12(100%)	CAN	Advising the research team on the design, conduct, or implementation of a haemodialysis trial.	
**Nielsen (2020)**	Workshops	8 (28)	DK	User involvement in development of telehealth intervention to improve transplant.	
**Nierse (2012)**	Co-researchers<br>Focus groups<br>Interviews	2 (33)<br>^<br>27	NL	Collaboration and co-ownership in research.	
**O'Lone (2020)**	Workshop<br>Co-researcher	8(14%)<br>^	AUS	Attending workshop, contributing feedback on workshop program and report.	Yes
**Odgers (2018)**	2/83 studies → CKD<br>1. Chesney – None<br>2. Langman – Workshop	0<br>^	INT	Research priority setting in childhood chronic disease.	
**Paterson (2010)**	Co-researcher<br>Community Advisory Committee	1<br>8 (73)	CAN	Toolkit for Aboriginal people on HD.	

**Patzer (2014)**	Steering committee	^	US	Reducing disparities in access to Tx.	Yes
**Perry (2022)**	Workshops<br>Stakeholder Meetings	^<br>^	US	Inform best practice into clinical workflows to reduce the burden of chronic disease on patients and health care system.	Yes
**Radisic (2022)**	Interviews<br>Co-researcher	25(100%)<br>3 (^)	AUS	Involved in developing and executing interviews. involving with preparing manuscript and publishing.	Yes
**Rheault (2020)**	Focus Groups<br>Patient Advocacy Groups	^	INT	Took part in panel discussions and breakout groups to accelerate the current research efforts in order to deliver the unmet needs of patients living with Alport syndrome.	
**Rosaasen (2018)**	Co-researcher<br>Consultants	1 (11)<br>8 (40)	CAN	Patient-oriented research project to improve patient education.	
**Rossum (2020)**	Focus Groups<br>Interview<br>Survey<br>Workshop<br>Patient Partners	65 (68%)<br>17 (55%)<br>228 (82%)<br>18 (47%)<br>4 (20%)	CAN	A mixed method investigation to determine priorities for improving information, interaction, and individualization of care among individuals on in-center haemodialysis.	Yes
**Schipper (2011)**	Interviews<br>Focus groups<br>Patient advocacy groups	20 (100)<br>54 (100)<br>^	NL	Priorities for social science research for patients on dialysis	
**Scholes-Robertson (2020)**	Commissioner / Proposer	1 (100%)	AUS	Manuscript preparation and publishing	
**Scholes-Robertson (2022a)**	semi-structured interviews<br>Commissioner / Proposer	18 (100%)<br>1	AUS	Consumer involvement in Interview-based study, Consumer lead-author.	

**Scholes-Robertson (2022b)**	Workshop<br>Commissioner	24 (57%)<br>1 (100%)	AUS	Participated in three workshops<br>Driver of research, manuscript preparation and submission.	
**Soondergard (2023)**	Commissioner / Proposer of research	1 (100%)	DK	Manuscript preparation and publishing	
**Tong (2012)**	Consumer advisor<br>Workshops	1<br>23 (100)	AUS	Consumer involvement in topic and outcome selection for guidelines.	Yes
**Tong (2015a)**	Workshop	30 (52)	AUS	Research priorities in CKD.	
**Tong (2015b)**	Of 16 studies identified, 4 explicitly involved patients:<br>1. Manns<br>2. Schipper<br>3. Rys-Sikora - online public consultation/voting<br>4. Tong - focus groups	 <br>**<br>**<br>^ (7)<br>63 (100)	 <br><br>US<br>AUS	Research priority setting.	
**Tong (2016)**	3 focus groups	18 (100)	AUS	Consumer priorities for guidelines ADPKD.	Yes
**Tong (2018)**	Workshop	6 (7)	INT	Implementing core outcomes in kidney disease.	Yes
**Tuttle (2021)**	Co-Researcher<br>Advisory Groups	1 (25)<br>^	US	Manuscript preparation and publishing.	
**Van der Horst (2022)**	Focus Group Participation<br>Working group.	8 (100%)<br>^	NL	Identify needs and inform the development, design, and usability of a clinic CKD dashboard.	
**Vanstone (2023)**	Patient Partner	1 (100%)	CAN	Manuscript preparation and publishing.	

**Vargas (2008)**	Workgroup<br>Delphi survey<br>Conference<br>Survey	^<br>^<br>25 (10)<br>^	US	Awareness, prevention, early intervention.	
**Walklin (2023)**	Survey<br>Consumer Advisory Group (PPI Group)	340 (100%)<br>6 (^)	UK	Involved in co-developing Kidney BEAM, but also early in the ethical approval stages. Consumers contributed to the writing of lay summaries, providing patient perspectives on data collection procedures, ethical issues, and trial dissemination plans.	Yes
**Ward (2018)**	Consumer advisory councils<br>Co-researcher	^<br>1	UK	Patient/ caregiver involvement in RCT.	
**Willis (2021)**	Interviews<br>Focus Groups	28 (100%)<br>17 (100%)	USA	Involved in the preparation, development and participation of the interviews and focus groups.	
**Wilson (2022)**	Expert Advisory Group<br>Co-researcher	2 (12)<br>2 (12)	UK	To ensure that the preintervention assessment of the study is appropriate for a kidney disease population.	

* % of group made up of consumer; **Reported in included study; NA = Not applicable; ^ = Not reported; JLA PSP=James Lind Alliance – Priority Setting Partnership, P=patient, US=United States, CAN=Canada, UK=United Kingdom, SE=Sweden, AU=Australia, KR=Korea, FR=France, IE=Ireland, DK=Denmark, INT=International, NL=Netherlands, GUAT = Guatemala

Supplementary Table 4. Definitions of terms relating to consumer involvement

Term	Definition
**Community-based participatory research**	"A collaborative approach to research that equitably involves all partners in the research process and recognizes the unique strengths that each brings." [66, 90, 115]
**Patient-researcher partnership**	"The mode in which patients are engaged in research, which implies that each partner contributes something of equal value to the common enterprise. It encompasses more than having patients engaged as study participants. Furthermore, it is important to make a distinction between patients as research partners and patients as participants in surveys or focus groups. In the latter, patients are participants, and their feedback and discussions serve to answer the research question. In patient-research partnerships, patients' input is sought to direct the various phases (preparatory, execution, and translational) of the research project."[51]
**Patient engagement**	"Patients having a "meaningful and active collaboration in governance, priority setting, conducting research and knowledge translation."[71]<br>"Collaborative research done by, with, and for patients to inform health care and health research decisions and questions."[51]<br>"The establishment of a relationship between patients and researchers and is the first step of involvement."[62]<br>"Reinforcing a shared purpose (learning together, collective commitment, evolving attitudes)"[42]<br>"Fostering a culture of responsive and innovative research (accessible supports, strengthened process and product)"[42]<br>"Aligning priorities, goals, and needs (amenability to patient involvement, mutually productive relationships, harmonizing expectations)"[42]
**Patient/consumer/public**	"A patient, caregiver or family member with lived experience of chronic kidney disease"[62]<br>"Fredriksson and Tritter (2017) [10] make the distinction between patient and public in PPI. Firstly, people with direct experience of health conditions either themselves or through a member of the family and secondly, people who have a more general interest in health and bring a 'public' view to health research."[43]<br>"An overarching term which includes those with personal health concerns and their friends, family and other informal caregivers who together engage with health and organizational systems"[121]
**Involvement**	"Research that is carried out "with or by" members of the public."[45]<br>"A sustained and meaningful contribution to the research process as more than a research subject or participant and can range from consultation to partnership."[62]

	“Research being carried out ‘with’ or ‘by’ members of the public rather than ‘to’, ‘about’ or ‘for’ them”[43]
**Co-researcher**	“Equal partners of the research team and carry out some or all of the research activities alongside or independent of the academic researchers.”[43]
**Co-production**	“The published NIHR INVOLVE guidance of research coproduction cites the key co-production principles as sharing of power, inclusion of perspectives, respecting the value and knowledge of all contributors, reciprocity, and the building of relationships”[118]
**Patient partner**	“Refers to individuals with personal experience of kidney disease or impacted by kidney disease (e.g., informal caregivers, family, friends, living kidney donors) who engage in network research partnerships and/or governance”[41, 59]<br>"People with lived experience who have longitudinal, bidirectional involvement with health organizations for the purpose of system change"[121]<br>“Patients with personal experience of dialysis or a family member who had experience supporting a patient receiving hemodialysis, who have been actively involved in discussions to advise a research team on the design, conduct, or implementation of a hemodialysis trial”[74]

Supplementary Table 5 – Guidance for Reporting Involvement of Patients and Public (GRIPP) 2: Short-form checklist

Section and topic	Item	Reported on page No
1: Aim	Report the aim of the study	5
2: Methods	Provide a clear description of the methods used for PPI in the study	6
3: Results	Outcomes – report the results of PPI in the study including positive and negative outcomes.	6-7
4: Discussion and Conclusions	Outcomes – comment on the extent to which PPI influenced the study overall. Describe positive and negative effects	15 - 18
5: Reflections/critical perspective	Comment critically on the study, reflecting on the things that went well and those that did not so that others can learn from this experience	15 - 18

PRISMA 2020 Checklist

Section and Topic	Item #	Checklist item	Location where item is reported
**TITLE**			
Title	1	Identify the report as a systematic review.	Page 2
**ABSTRACT**			
Abstract	2	See the PRISMA 2020 for Abstracts checklist.	Page 4
**INTRODUCTION**			
Rationale	3	Describe the rationale for the review in the context of existing knowledge.	Page 6
Objectives	4	Provide an explicit statement of the objective(s) or question(s) the review addresses.	Page 6-7
**METHODS**			
Eligibility criteria	5	Specify the inclusion and exclusion criteria for the review and how studies were grouped for the syntheses.	Page 7
Information sources	6	Specify all databases, registers, websites, organisations, reference lists and other sources searched or consulted to identify studies. Specify the date when each source was last searched or consulted.	Page 7
Search strategy	7	Present the full search strategies for all databases, registers and websites, including any filters and limits used.	Page 7
Selection process	8	Specify the methods used to decide whether a study met the inclusion criteria of the review, including how many reviewers screened each record and each report retrieved, whether they worked independently, and if applicable, details of automation tools used in the process.	Page 7-8
Data collection process	9	Specify the methods used to collect data from reports, including how many reviewers collected data from each report, whether they worked independently, any processes for obtaining or confirming data from study investigators, and if applicable, details of automation tools used in the process.	Page 7-8
Data items	10a	List and define all outcomes for which data were sought. Specify whether all results that were compatible with each outcome domain in each study were sought (e.g. for all measures, time points, analyses), and if not, the methods used to decide which results to collect.	Page 7-8
	10b	List and define all other variables for which data were sought (e.g. participant and intervention characteristics, funding sources). Describe any assumptions made about any missing or unclear information.	Page 7-8
Study risk of bias assessment	11	Specify the methods used to assess risk of bias in the included studies, including details of the tool(s) used, how many reviewers assessed each study and whether they worked independently, and if applicable, details of automation tools used in the process.	Page 7-8
Effect measures	12	Specify for each outcome the effect measure(s) (e.g. risk ratio, mean difference) used in the synthesis or presentation of results.	N/A
Synthesis methods	13a	Describe the processes used to decide which studies were eligible for each synthesis (e.g. tabulating the study intervention characteristics and comparing against the planned groups for each synthesis (item #5)).	Page 8-9
	13b	Describe any methods required to prepare the data for presentation or synthesis, such as handling of missing summary statistics, or data conversions.	Page 8-9
	13c	Describe any methods used to tabulate or visually display results of individual studies and syntheses.	Page 8-9
	13d	Describe any methods used to synthesize results and provide a rationale for the choice(s). If meta-analysis was performed, describe the model(s), method(s) to identify the presence and extent of statistical heterogeneity, and software package(s) used.	Page 8-9
	13e	Describe any methods used to explore possible causes of heterogeneity among study results (e.g. subgroup analysis, meta-regression).	N/A
	13f	Describe any sensitivity analyses conducted to assess robustness of the synthesized results.	N/A
Reporting bias assessment	14	Describe any methods used to assess risk of bias due to missing results in a synthesis (arising from reporting biases).	N/A
Certainty assessment	15	Describe any methods used to assess certainty (or confidence) in the body of evidence for an outcome.	N/A

PRISMA 2020 Checklist

Section and Topic	Item #	Checklist item	Location where item is reported
**RESULTS**			
Study selection	16a	Describe the results of the search and selection process, from the number of records identified in the search to the number of studies included in the review, ideally using a flow diagram.	Page 8
	16b	Cite studies that might appear to meet the inclusion criteria, but which were excluded, and explain why they were excluded.	Page 8
Study characteristics	17	Cite each included study and present its characteristics.	Page 8 -15
Risk of bias in studies	18	Present assessments of risk of bias for each included study.	N/A
Results of individual studies	19	For all outcomes, present, for each study: (a) summary statistics for each group (where appropriate) and (b) an effect estimate and its precision (e.g. confidence/credible interval), ideally using structured tables or plots.	N/A
Results of syntheses	20a	For each synthesis, briefly summarise the characteristics and risk of bias among contributing studies.	N/A
	20b	Present results of all statistical syntheses conducted. If meta-analysis was done, present for each the summary estimate and its precision (e.g. confidence/credible interval) and measures of statistical heterogeneity. If comparing groups, describe the direction of the effect.	N/A
	20c	Present results of all investigations of possible causes of heterogeneity among study results.	N/A
	20d	Present results of all sensitivity analyses conducted to assess the robustness of the synthesized results.	N/A
Reporting biases	21	Present assessments of risk of bias due to missing results (arising from reporting biases) for each synthesis assessed.	N/A
Certainty of evidence	22	Present assessments of certainty (or confidence) in the body of evidence for each outcome assessed.	N/A
**DISCUSSION**			
Discussion	23a	Provide a general interpretation of the results in the context of other evidence.	Page 15-19
	23b	Discuss any limitations of the evidence included in the review.	Page 17-18
	23c	Discuss any limitations of the review processes used.	Page 17-18
	23d	Discuss implications of the results for practice, policy, and future research.	Page 18-19
**OTHER INFORMATION**			
Registration and protocol	24a	Provide registration information for the review, including register name and registration number, or state that the review was not registered.	N/A
	24b	Indicate where the review protocol can be accessed, or state that a protocol was not prepared.	N/A
	24c	Describe and explain any amendments to information provided at registration or in the protocol.	N/A
Support	25	Describe sources of financial or non-financial support for the review, and the role of the funders or sponsors in the review.	Page 19
Competing interests	26	Declare any competing interests of review authors.	Page 19
Availability of data, code and other materials	27	Report which of the following are publicly available and where they can be found: template data collection forms; data extracted from included studies; data used for all analyses; analytic code; any other materials used in the review.	Page 20

From: Page MJ, McKenzie JE, Bossuyt PM, Boutron I, Hoffmann TC, Mulrow CD, et al. The PRISMA 2020 statement: an updated guideline for reporting systematic reviews. BMJ 2021;372:n71. doi: 10.1136/bmj.n71
